# A Positive Relationship Exists between the Triglyceride to Glucose Index and Waist-to-Hip Ratio with Stroke Risk in Middle-Aged and Older Chinese

**DOI:** 10.1523/ENEURO.0264-25.2025

**Published:** 2025-11-26

**Authors:** Aihua Chen, Cishuang Fu, Haiying Chen, Wei Peng, Yangchen Ou, Qin Guo, Mingyan Xie

**Affiliations:** ^1^Departments of General Medicine, Changde Hospital, Xiangya School of Medicine, Central South University (The First People's Hospital of Changde City), Changde 415000, China; ^2^Gastroenterology, Changde Hospital, Xiangya School of Medicine, Central South University (The First People's Hospital of Changde City), Changde 415000, China

**Keywords:** CHARLS, middle-aged and elderly, nonlinear association, smooth curve fitting, stroke, triglyceride–glucose–waist-to-hip ratio (TyG–WHR)

## Abstract

This study determined the association between the triglyceride-glucose (TyG) index–waist-to-hip ratio (TyG–WHR) and stroke. Data from the China Health and Retirement Longitudinal Study (CHARLS) were utilized from baseline in 2011 to the wave six follow-up in 2020. The CHARLS cohort was assembled using a multistage probability sampling technique. Participants were comprehensively assessed through standardized questionnaires with face-to-face interviews. A total of 4,911 patients with 2,338 males (47.6%) and 2,573 females (52.4%) were included in this analysis. A significant association between the TyG–WHR and the risk of stroke was identified utilizing a Cox proportional hazards regression model with cubic spline functions that were characterized by a nonlinear relationship. The analysis determined a threshold for the TyG–WHR at 4.635. The association between the TyG–WHR and stroke was not significant [hazard ratio (HR), 0.813; 95% CI, 0.662–0.999; *p* = 0.049] to the left of the threshold. The association was statistically significant (HR, 1.271; 95% CI, 1.131–1.429; *p* < 0.001) to the right of the threshold. The current study demonstrated a positive and nonlinear association between the TyG–WHR and stroke risk among middle-aged and elderly Chinese populations. When the TyG–WHR exceeded 4.635, there was a statistically significant positive correlation with the occurrence of stroke. Clinically, reducing the TyG–WHR, especially <4.635, may reduce the risk of stroke.

## Significance Statement

This study determined the association between the triglyceride–glucose–waist-to-hip ratio (TyG–WHR) and stroke from CHARLS database. The current study demonstrated a positive and nonlinear association between the TyG–WHR and stroke risk among middle-aged and elderly Chinese populations. When the TyG–WHR exceeded 4.635, there was a statistically significant positive correlation with the occurrence of stroke. Clinically, reducing the TyG–WHR, especially <4.635, may reduce the risk of stroke. The study focused on the clinical risk factor for stroke, which is commonly discussed in the population health around the world.

## Introduction

Stroke occurs in ∼15 million people worldwide each year with one-third of these patients dying and another one-third becoming permanently disabled, according to the World Health Organization (Rudnicka et al., 2020). The incidence of stroke in the middle-aged and elderly population has further increased with the aging population. The incidence of stroke is particularly prominent in China, where studies in recent years have shown that the annual incidence of stroke in people ≥45 years of age is ∼250 cases per 100,000 (Rudnicka et al., 2020). Stroke has ascended to the position of the third most significant factor in the worldwide burden of disease, surpassing its previous ranking of fifth place in recent times (GBD 2019 Stroke Collaborators, 2021). As a major health problem, stroke severely impairs quality of life and generates a heavy economic and social burden, especially in low-income countries (GBD 2019 Stroke Collaborators, 2021). Therefore, it is imperative to effectively identify and manage modifiable risk factors to reduce stroke incidence and mitigate its broader impact.

Insulin resistance (IR) is an independent risk factor for stroke and influences the patient's prognosis (Ding et al., 2022). However, direct measurement of IR in clinical settings is complex and not routinely feasible. The triglyceride–glucose (TyG) index is a parameter consisting of fasting triglycerides (TG) and fasting plasma glucose (FPG), which has been confirmed in studies to be a reliable assessment of IR (Guerrero-Romero et al., 2010; Han et al., 2023). The TyG index is an easily obtained surrogate marker for IR and can represent the crucial development of stroke. A significant association has been reported between a high TyG index and adverse poststroke outcomes, such as high mortality or stroke recurrence (Yang et al., 2023). Zhao et al. reported that an elevated TyG index is the independent risk factor for stroke patients (Zhao et al., 2021). A cutoff TyG index ≥8.41 should be considered a criterion for screening high-risk stroke populations (Xu et al., 2023).

According to a statistical report by the Global Burden of Diseases, Injuries, and Risk Factors Study, a high body-mass index (BMI) was the fastest-growing risk factor for stroke from 1990 to 2019 (GBD 2019 Stroke Collaborators, 2021). The waist-to-hip ratio (WHR) is closely related to the BMI. It has been recently reported that the new parameters [TyG–BMI, TyG–waist circumference (TyG–WC) ratio, TyG–WHR, and TyG–waist-to-height ratio (TyG–WHtR)] have a better performance in the assessment of IR than the TyG index (Lim et al., 2019). Combining the TyG with obesity metrics and analyzing these metrics in relation to hypertension and cardiovascular risk has shown that the TyG–WHR has the highest efficacy in differentiating between individuals at high cardiovascular risk (H. Miao et al., 2024). The TyG–WHR index represents the collaborative impact of IR and central fat distribution, two closely linked mechanisms fundamental to the etiology of metabolic and vascular disorders (Y. Miao et al., 2024; Yadegar et al., 2025). The improved predictive accuracy could be ascribed to the index's capacity to combine two distinct pathophysiological mechanisms: IR, fostering systemic metabolic dysregulation and endothelial strain, and central adiposity, contributing to localized inflammatory and atherogenic alterations. Their combined impact may offer a more holistic representation of cardiovascular and cerebrovascular susceptibility. As an emerging metabolic metric, the TyG–WHR is strongly associated with the prevalence and poor prognosis of a variety of metabolic diseases, including Type 2 diabetes and cardiovascular disease (Yang et al., 2024; Zhang et al., 2024). Given the substantial evidence indicating positive associations between the TyG index and WHR and stroke, we hypothesized that the TyG–WHR might positively correlate with stroke risk. However, the relationship between the TyG–WHR and stroke has been less frequently reported. Nonetheless, its association with stroke, especially in prospective cohorts, has not been thoroughly investigated.

Our hypothesis posited that TyG–WHR might function as a valuable predictor of stroke. We conducted a prospective cohort study utilizing data from the China Health and Retirement Longitudinal Study (CHARLS) to investigate the correlation between TyG–WHR and the incidence of stroke. The primary objective of this study is to assess the potential of TyG–WHR as a stand-alone predictor of stroke and to provide insights for primary prevention strategies.

## Materials and Methods

### Study population

The current study was based on data from the CHARLS, a comprehensive national cohort study designed to assess the economic, social, and health conditions of the population (Zhao et al., 2014). The CHARLS utilized stratified sampling and probability proportional to size sampling methods to conduct longitudinal surveys in 450 communities/villages across 150 counties in China, tracking households and individuals ≥45 years of age. Therefore, the CHARLS data are highly representative and accurately reflect the overall situation of the urban and rural elderly populations in China.

**Figure 1.**
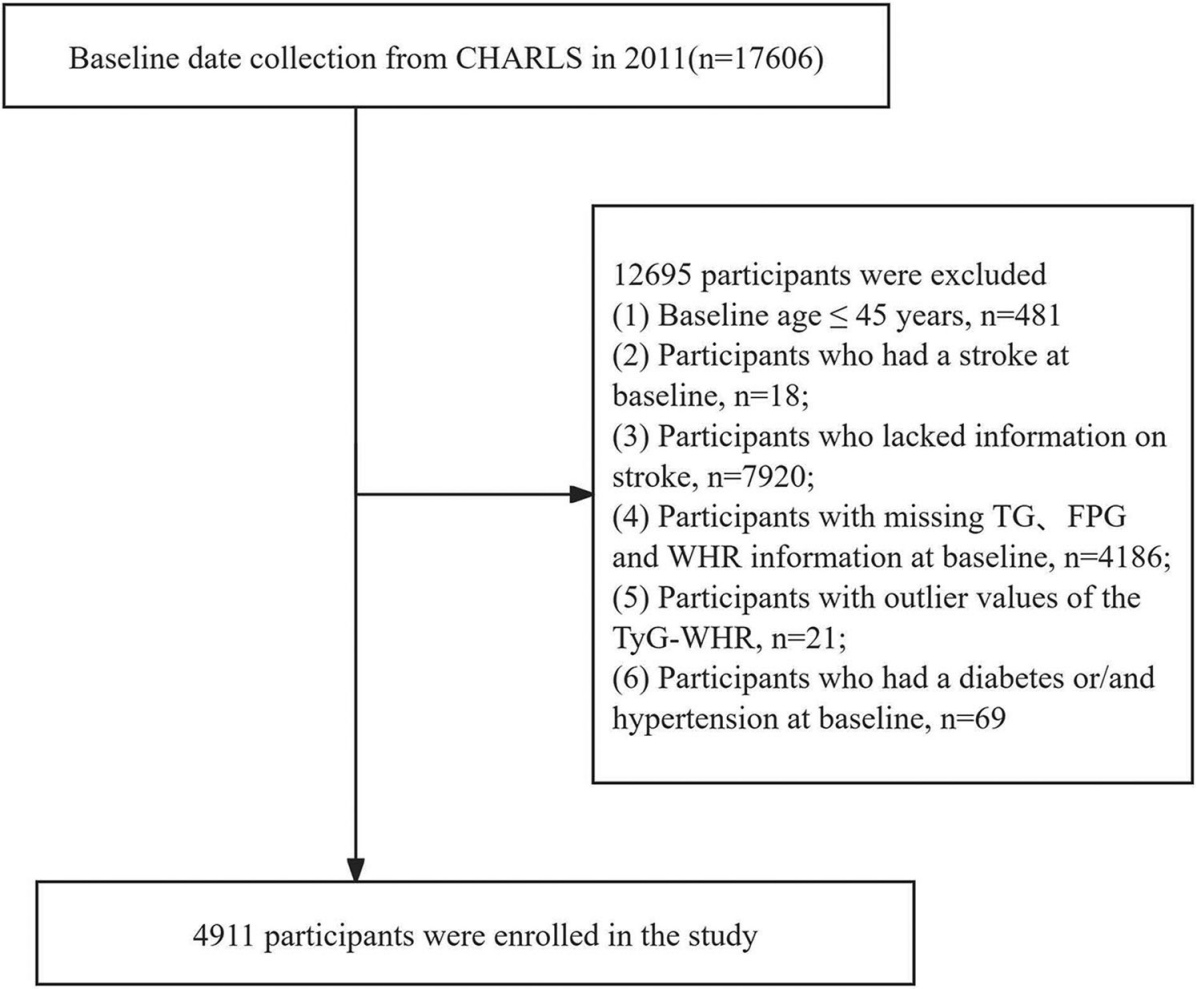
Flowchart of patient inclusion. CHARLS, China Health and Retirement Longitudinal Study; TG, triglycerides; FPG, fasting blood glucose; TyG–WHR, triglyceride–glucose–waist-to-hip ratio.

A 9 year longitudinal study (2011–2020) was conducted using the 2011 population data as the baseline. TyG–WHR was used as the baseline exposure variable for analysis, and whether a stroke occurred in 2020 was taken as the outcome variable, with an intermediate follow-up of 9 years. In this study, 17,606 participants who had undergone blood testing were reviewed. Participants were excluded as follows: (1) 4,186 participants with missing TG, FPG, and WHR data at the baseline; (2) 18 participants who had a stroke at baseline; (3) 7,920 participants who lacked information on stroke; (4) 69 participants who had diabetes or/and hypertension at the baseline; and (5) 21 participants with TyG–WHR values exceeding three standard deviations from the mean. After applying these criteria, 4,911 participants were included in the study, with 2,338 males (47.6%) and 2,573 females (52.4%). The detailed methodology of the participant selection process is depicted in Figure 1.

This study received ethical approval from the Biomedical Ethics Review Committee of our University. All study participants signed an informed consent form related to this study prior to inclusion. Researchers interested in accessing and downloading the study data and relevant information can do so via the official CHARLS project website (http://charls.pku.edu.cn/).

### Variables

#### Assessment of TyG–WHR changes

The FBG and TG levels were obtained from blood sample testing performed at the baseline survey in 2011. FBG and TG levels were not measured and applied to analysis during the follow-up period. The specific procedure for defining the TyG–WHR in this study was as follows: TyG–WHR = TyG index × WHR, where the TyG index = ln [FPG (mg/dl) × TG (mg/dl)/2; Guerrero-Romero et al., 2010; H. Miao et al., 2024]. The WHR was determined by dividing WC by hip circumference (HC). WC was assessed at the midpoint between the lower margin of the last palpable rib and the iliac crest, and HC was measured at the widest part of the buttocks, with participants standing upright and arms relaxed at their sides.

According to the TyG–WHR calculated, the population were classified into four groups: Q1 < 4.79, 4.79 ≤ Q2 < 5.45, 5.45 ≤ Q3 < 6.22, and Q4 ≥ 6.22.

### Diagnosis of stroke

The outcome variable in this study was incident stroke during follow-up. Incident cases were identified as participants who did not have a stroke at the start of the study and subsequently reported experiencing a stroke during the follow-up assessment, as previously described (Wang et al., 2021). Data on stroke occurrence were carefully collected through a structured self-report questionnaire to obtain comprehensive information in three key areas: (1) Were you informed of your stroke diagnosis by a healthcare professional? (2) When did you initially receive or become aware of the diagnosis? (3) Are you currently receiving follow-up treatment related to stroke? If a participant answered “yes” to these questions during follow-up, he/she was categorized as receiving a stroke diagnosis for the first time, and the self-reported time was recorded as the onset of the stroke. The timing of the stroke event was calculated by subtracting the baseline survey time from the stroke onset time. For participants who did not report a stroke during the follow-up period, the follow-up time was determined by the interval between the baseline assessment and the final investigation date.

### Assessment of covariates

Sociodemographic characteristics included age, gender, marital status, educational level (elementary or below and junior high school and above), and geographic location (rural or urban). Lifestyle factors included cigarette smoking status, alcohol consumption status, and physical activity level. Health conditions included weight, systolic blood pressure (SBP), diastolic blood pressure (DBP), hypertension, and diabetes. Laboratory examinations included FPG, TG, total cholesterol, high-density lipoprotein–cholesterol (HDL–C), and low-density lipoprotein–cholesterol (LDL–C). Measurements included weight, height, WC, and HC. The WHR was the WC-to-HC ratio. SBP and DBP were measured using a standard mercury sphygmomanometer.

### Statistical analysis

In this study, continuous variables are expressed as the mean ± standard deviation or median (interquartile range). Categorical variables are expressed as frequencies or percentages.

The association between the TyG–WHR and stroke events was determined in three steps. In Step 1, three models were constructed using univariate and multivariate linear regression analyses. Model 1 did not incorporate any covariates. Model 2 adjusted for age, gender, marital status, educational level, and geographic location. Model 3 adjusted for additional covariates listed in Table 1 based on Model 2. The purpose of constructing these models was to determine the trend of the TyG–WHR effect values under different adjustment strategies and to ascertain the robustness of the results. In Step 2, the potential nonlinear relationship between the TyG–WHR and stroke events was addressed. The Cox proportional risk model was used for smooth curve fitting. When nonlinearity was observed, recursive algorithms were used to determine the inflection point. A segmented Cox proportional risk model was then formulated on either side of the inflection points. The optimal model for the relationship between the TyG–WHR and stroke risk was determined using the log-likelihood ratio test. In Step 3, subgroup analyses were performed using stratified linear regression or generalized additive models. If the stratified variables were continuous, the variables were converted to categorical variables by clinical cutoff or tertile and then tested for interactions. Effect modification tests for subgroup indicators were realized using likelihood ratio tests. Sensitivity analyses were performed to ensure the robustness of the data analysis. The TyG–WHR was converted into a categorical variable, and the *p* value for trend was calculated to verify the results of the TyG–WHR as a continuous variable and assess potential nonlinearity. Statistical analyses utilized the R software (version 4.2.3; R Foundation for Statistical Computing), with primary packages such as rms for regression modeling and restricted cubic splines, survival for Cox regression, and ggplot2 for data visualization. Statistical significance was defined as *p* < 0.05 (two-sided).

**Table 1.** Baseline characteristics of participants

Indicators/TyG–WHR quartile	Total	Q1 < 4.79	4.79 ≤ Q2 < 5.45	5.45 ≤ Q3 < 6.22	Q4 ≥ 6.22	*p* value
*N*	4,911	1,232	1,229	1,225	1,225	
Age (year)	59.86 (9.32)	59.59 (9.36)	59.42 (9.27)	60.36 (9.38)	60.04 (9.26)	0.053
Gender						<0.001*
Male	2,338 (47.61%)	628 (50.97%)	646 (52.56%)	533 (43.51%)	531 (43.35%)	
Female	2,573 (52.39%)	604 (49.03%)	583 (47.44%)	692 (56.49%)	694 (56.65%)	
Marital						0.299
Nonmarried	794 (16.17%)	195 (15.83%)	213 (17.33%)	206 (16.82%)	180 (14.69%)	
Married	4,117 (83.83%)	1,037 (84.17%)	1,016 (82.67%)	1,019 (83.18%)	1,045 (85.31%)	
Location						0.003*
Rural	4,413 (89.86%)	1,128 (91.56%)	1,120 (91.13%)	1,093 (89.22%)	1,072 (87.51%)	
Urban	498 (10.14%)	104 (8.44%)	109 (8.87%)	132 (10.78%)	153 (12.49%)	
Education						0.547
Elementary or below	3,469 (70.64%)	889 (72.16%)	859 (69.89%)	866 (70.69%)	855 (69.80%)	
Junior high school and above	1,442 (29.36%)	343 (27.84%)	370 (30.11%)	359 (29.31%)	370 (30.20%)	
Physical activity level						0.387
Inactive	591 (12.03%)	153 (12.42%)	142 (11.55%)	127 (10.37%)	169 (13.80%)	
Minimally active	1,482 (30.18%)	368 (29.87%)	377 (30.68%)	382 (31.18%)	355 (28.98%)	
Moderately active	1,585 (32.27%)	385 (31.25%)	397 (32.30%)	398 (32.49%)	405 (33.06%)	
Vigorously active	1,253 (25.51%)	326 (26.46%)	313 (25.47%)	318 (25.96%)	296 (24.16%)	
Cigarette smoking						<0.001*
Never	2,878 (58.60%)	667 (54.14%)	669 (54.43%)	755 (61.63%)	787 (64.24%)	
Current smoker	1,870 (38.08%)	530 (43.02%)	519 (42.23%)	422 (34.45%)	399 (32.57%)	
Former smoker	163 (3.32%)	35 (2.84%)	41 (3.34%)	48 (3.92%)	39 (3.18%)	
Alcohol consumption						<0.001*
Never	3,315 (67.50%)	793 (64.37%)	787 (64.04%)	871 (71.10%)	864 (70.53%)	
Current consumer	376 (7.66%)	126 (10.23%)	89 (7.24%)	77 (6.29%)	84 (6.86%)	
Former consumer	1,220 (24.84%)	313 (25.41%)	353 (28.72%)	277 (22.61%)	277 (22.61%)	
SBP (mmHg)	132.31 (25.58)	128.18 (24.14)	129.42 (23.08)	133.47 (23.01)	138.07 (30.24)	<0.001*
DBP (mmHg)	75.98 (12.97)	73.74 (12.65)	74.84 (12.31)	76.20 (11.59)	79.08 (14.55)	<0.001*
Weight (kg)	59.09 (11.70)	54.21 (10.31)	56.97 (9.58)	60.09 (11.01)	65.11 (12.73)	<0.001*
TyG index	2.22 (0.30)	1.99 (0.24)	2.14 (0.21)	2.27 (0.23)	2.49 (0.28)	<0.001*
WHR	2.48 (0.43)	2.09 (0.47)	2.42 (0.23)	2.59 (0.26)	2.83 (0.33)	<0.001*
TyG–WHR	5.50 (1.19)	4.10 (0.82)	5.13 (0.19)	5.81 (0.22)	6.99 (0.67)	<0.001*
FPG (mg/dl)	109.55 (35.29)	105.25 (28.00)	107.61 (33.16)	112.85 (41.02)	112.52 (37.13)	<0.001*
HDL–C (mg/dl)	50.97 (15.44)	56.82 (15.59)	53.27 (15.39)	49.73 (14.42)	44.01 (13.31)	<0.001*
LDL–C (mg/dl)	117.54 (35.64)	112.75 (32.58)	116.24 (34.92)	119.38 (36.04)	121.83 (38.20)	<0.001*
TG (mg/dl)	106.2 (75.22, 155.76)	70.80 (54.87, 95.80)	92.92 (73.45, 123.02)	117.70 (89.39, 158.41)	170.81 (125.67, 257.54)	<0.001*

TyG–WHR, triglyceride–glucose–waist-to-hip ratio; *N*, number; SBP, systolic blood pressure; DBP, diastolic blood pressure; TyG, triglyceride–glucose; WHR, waist-to-hip ratio; FPG, fasting blood glucose; HDL–C, high-density lipoprotein–cholesterol; LDL–C, low-density lipoprotein–cholesterol; TG, triglycerides.

* *p* < 0.05.

## Results

### Study population characteristics

A total of 4,911 patients were included in this study (Fig. 1). Table 1 displays the baseline characteristics categorized into quartiles based on the TyG–WHR. A total of 4,911 participants (2,338 males and 2,573 females) with a mean age of 59.86 years (±9.32 years) participated in the analysis. The results showed that various parameters, including SBP, DBP, weight, TyG, WHR, FPG, TG, and LDL–C, increased significantly with increasing TyG–WHR values. In contrast, HDL–C showed opposite trends. Furthermore, the proportion of noncigarette smokers, nonconsumers of alcohol, females, and urban residents increased as the TyG–WHR increased, whereas the proportion of males gradually decreased. The education level and marital status showed no significant differences among the quartiles (Table 1).

A stratified analysis was conducted using this threshold to investigate further the relevance of the identified inflection point (TyG–WHR = 4.635). Participants were categorized into TyG–WHR < 4.635 and TyG–WHR ≥ 4.635. The baseline characteristics of the groups are detailed in Table 2. The high TyG–WHR group exhibited significantly higher levels of SBP (133.34 vs 127.89 mmHg), DBP (76.58 vs 73.43 mmHg), FPG (110.67 vs 104.96 mg/dl), LDL–C, TG, and TyG index. Moreover, the high TyG–WHR group demonstrated increased body weight and WHR. A notably lower proportion of HDL–C was also noted in the higher TyG–WHR group. These disparities indicate distinct cardiometabolic profiles across the threshold and underscore the potential of TyG–WHR as a clinically relevant marker.

**Table 2.** Baseline characteristics of participants stratified by the TyG–WHR inflection point (4.635)

TyG–WHR quartile	<4.635	≥4.635	*p* value
N	965	3,946	
Age (year)	59.75 (9.52)	59.88 (9.28)	0.714
SBP (mmHg)	127.89 (24.92)	133.34 (25.62)	<0.001*
DBP (mmHg)	73.43 (12.67)	76.58 (12.97)	<0.001*
FPG (mg/dl)	104.96 (26.94)	110.67 (36.96)	<0.001*
HDL–C (mg/dl)	57.37 (15.90)	49.40 (14.92)	<0.001*
LDL–C (mg/dl)	112.42 (32.63)	118.80 (36.24)	<0.001*
TG (mg/dl)	82.62 (58.70)	145.40 (106.42)	<0.001*
TyG	1.97 (0.25)	2.28 (0.28)	<0.001*
Weight (kg)	54.03 (10.50)	60.32 (11.63)	<0.001*
WHR	2.03 (0.50)	2.59 (0.33)	<0.001*
TyG–WHR	3.92 (0.85)	5.89 (0.90)	<0.001*
Gender			0.001*
Male	504 (52.23%)	1,834 (46.48%)	
Female	461 (47.77%)	2,112 (53.52%)	
Location			0.126
Rural	880 (91.19%)	3,533 (89.53%)	
Urban	85 (8.81%)	413 (10.47%)	
Education			0.562
Elementary or below	689 (71.40%)	2,780 (70.45%)	
Junior high school and above	276 (28.60%)	1,166 (29.55%)	
Marital			0.847
Nonmarried	158 (16.37%)	636 (16.12%)	
Married	807 (83.63%)	3,310 (83.88%)	
Activities			0.719
Inactive	126 (13.06%)	465 (11.78%)	
Minimally active	285 (29.53%)	1,197 (30.33%)	
Moderately active	306 (31.71%)	1,279 (32.41%)	
Vigorously active	248 (25.70%)	1,005 (25.47%)	
Smoking			<0.001*
Never smoker	510 (52.85%)	2,368 (60.01%)	
Current smoker	430 (44.56%)	1,440 (36.49%)	
Former smoker	25 (2.59%)	138 (3.50%)	
Drinking			<0.001*
Never drinker	614 (63.63%)	2,701 (68.45%)	
Current drinker	102 (10.57%)	274 (6.94%)	
Former drinker	249 (25.80%)	971 (24.61%)	

*N*, number; SBP, systolic blood pressure, DBP, diastolic blood pressure; FPG, fasting blood glucose; HDL–C, high-density lipoprotein–cholesterol; LDL–C, low-density lipoprotein–cholesterol; TG, triglycerides; TyG, triglyceride–glucose; WHR, waist-to-hip ratio.

* *p* < 0.05.

### Incidence of stroke among the participants

**Figure 2.**
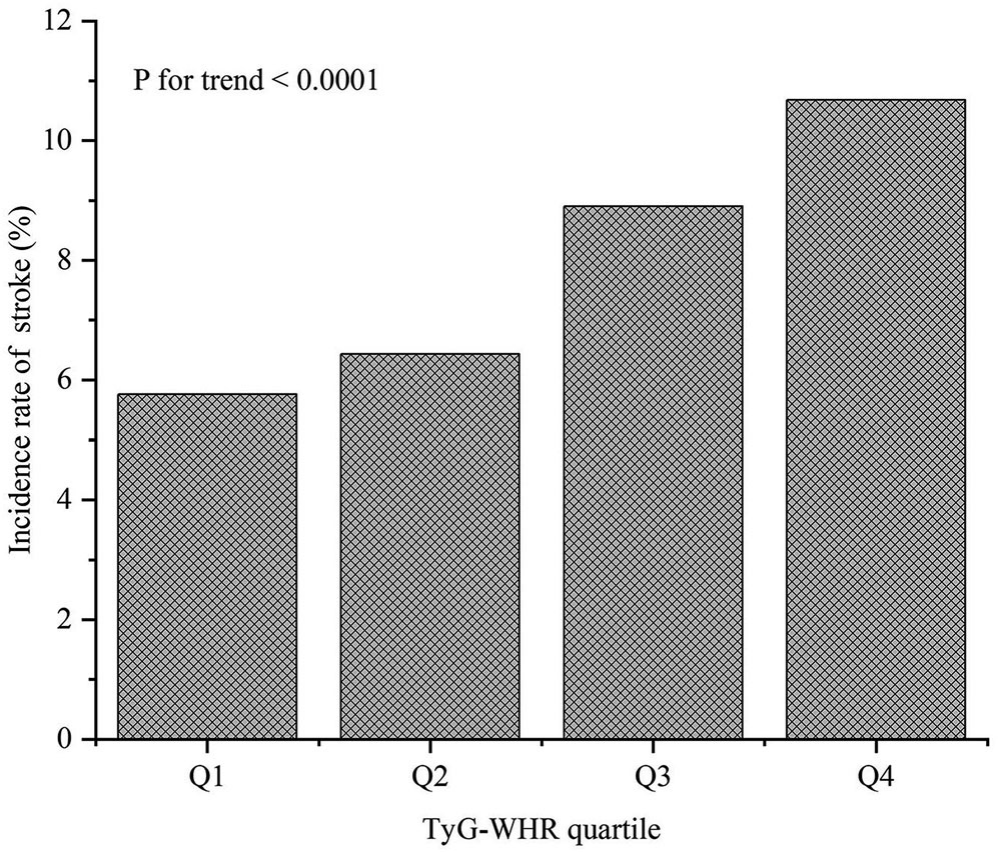
Incidence of stroke in each TyG–WHR quartile (*p* < 0.001 for trend). TyG–WHR, triglyceride–glucose–waist-to-hip ratio. Q means quartile grouping according to TyG–WHR, Q1 < 4.79, and 4.79 ≤ Q2 < 5.45; 5.45 ≤ Q3 < 6.22; Q4 ≥ 6.22.

Table 3 and Figure 2 show that 392 participants had a stroke. The incidences of stroke in each TyG–WHR quartile were as follows: Q1, 5.76%; Q2, 6.43%; Q3, 8.9%; and Q4, 10.86%. Participants with a lower TyG–WHR had a markedly lower stroke incidence than those with a higher TyG–WHR (*p* < 0.001).

**Table 3.** Incidence of stroke in participants

TyG–WHR (quartile)	Participants (*n*)	Stroke events (*n*)	Incidence rate (95% CI; %)
Total	4,911	392	7.98 (7.24–8.77)
Q1 (<4.79)	1,232	71	5.76 (4.56–7.17)
Q2 (4.79–5.45)	1,229	79	6.43 (5.16–7.91)
Q3 (5.45–6.22)	1,225	109	8.90 (7.40–10.59)
Q4 (≥6.22)	1,225	133	10.86 (9.06–12.49)
*p* for trend			<0.0001*

TyG–WHR, triglyceride–glucose–waist-to-hip ratio; CI, confidence interval.

* *p* < 0.05.

### Kaplan–Meier curve analysis

**Figure 3.**
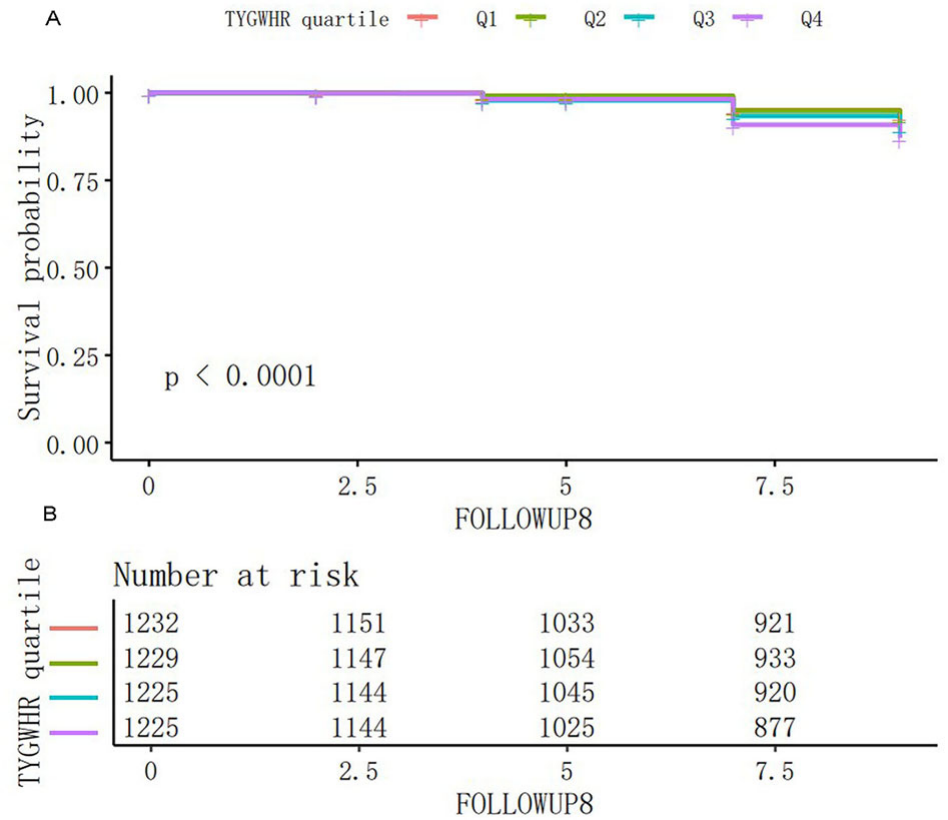
Kaplan–Meier analysis results. ***A***, Kaplan–Meier event-free survival curve. ***B***, Kaplan–Meier analysis of incident stroke based on four groups (log-rank, *p* < 0.001). TyG–WHR, triglyceride–glucose–waist-to-hip ratio. Q means quartile grouping according to TyG–WHR, Q1 < 4.79, and 4.79 ≤ Q2 < 5.45; 5.45 ≤ Q3 < 6.22; Q4 ≥ 6.22.

Figure 3 illustrates the Kaplan–Meier survival curves stratified by TyG–WHR quartiles for stroke-free survival. There were significant differences in the probability of stroke-free survival between the TyG–WHR quartiles (log-rank test, *p* < 0.001). Participants in the highest TyG–WHR quartile (Q4) demonstrated a significantly higher risk of stroke among patients with stroke.

### Relationship between the TyG–WHR and risk of stroke

**Figure 4.**
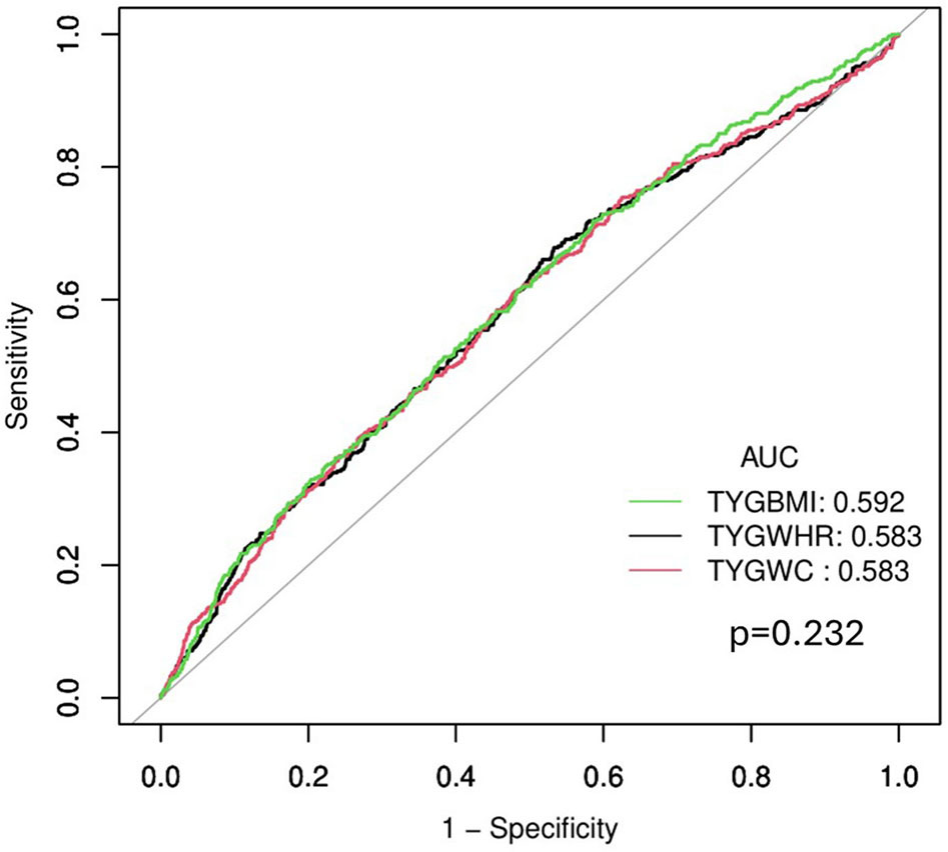
ROC curves comparing the performance of TyG–BMI, TyG–WHR, and TyG–WC in association with stroke. ROC, receiver operating characteristic; TyG, triglyceride–glucose; BMI, body-mass index; WHR, waist-to-hip ratio; WC, waist circumference.

To determine the association between the TyG–WHR and stroke risk, three Cox proportional hazards regression models were developed, as detailed in Table 4. The TyG–WHR exhibited a positive association with stroke. A one-unit increment in the TyG–WHR was associated with a 24% elevation in stroke risk in Model 1 [hazard ratio (HR), 1.24; 95% CI, 1.14–1.34; *p* < 0.001]. Model 2 demonstrated a 22% increase in stroke risk per one-unit increase in the TyG–WHR (HR,1.22; 95% CI, 1.12–1.33, *p* < 0.001). In Model 3, after accounting for all relevant factors, each one-unit incremental increase in the TyG–WHR was linked to a 13% increase in the risk of stroke (HR, 1.13; 95% CI, 1.03–1.25; *p* = 0.012). In addition, when assessed according to the TyG–WHR quartile classification, a significant 45% increase in stroke risk existed in the highest quartile (Q4) group compared with the lowest quartile (Q1) group (Model 3, HR,1.45; 95% CI, 1.04–2.02; *p* = 0.028). This trend remained robust in the progressively adjusted model (*p* < 0.05). To compare the discriminatory performance of TyG–WHR with other related indices for stroke, a receiver operating characteristic (ROC) analysis was conducted on TyG–WHR, TyG–BMI, and TyG–WC. Figure 4 shows that TyG–BMI yielded a slightly higher AUC of 0.592 than TyG–WHR and TyG–WC, which had an AUC of 0.583. However, these differences were not statistically significant (*p* = 0.232).

**Table 4.** Relationship between TyG–WR and the risk of stroke in different models

Variables	Model 1 (HR, 95% CI)	*p*	Model 2 (HR, 95% CI)	*p*	Model 3 (HR, 95% CI)	*p*
TyG–WHR	1.24 (1.14, 1.34)	<0.001*	1.22 (1.12, 1.33)	<0.001*	1.13 (1.03, 1.25)	0.012*
TyG–WHR quartile						
Q1	1 (Ref)		1 (Ref)		1 (Ref)	
Q2	1.10 (0.80, 1.51)	0.568	1.10 (0.80, 1.51)	0.569	1.04 (0.75, 1.44)	0.800
Q3	1.53 (1.14, 2.06)	0.005*	1.48 (1.10, 2.00)	0.010*	1.26 (0.92, 1.73)	0.143
Q4	1.91 (1.43, 2.55)	<0.001*	1.84 (1.38, 2.46)	<0.001*	1.45 (1.04, 2.02)	0.028*
*p* for trend		<0.001*		<0.001*		0.015*

Model 1 was a crude model. Model 2 was adjusted for age, gender, geographic location, education, marital status, and physical activity. Model 3 was adjusted for age, gender, geographic location, education, marital status, physical activity, cigarette smoking and alcohol consumption status, weight, SBP, DBP, HDL–C, and LDL–C. HR, hazard ratio; CI, confidence interval; Ref, reference.

* *p* < 0.05.

### Analysis of the nonlinear relationship

**Figure 5.**
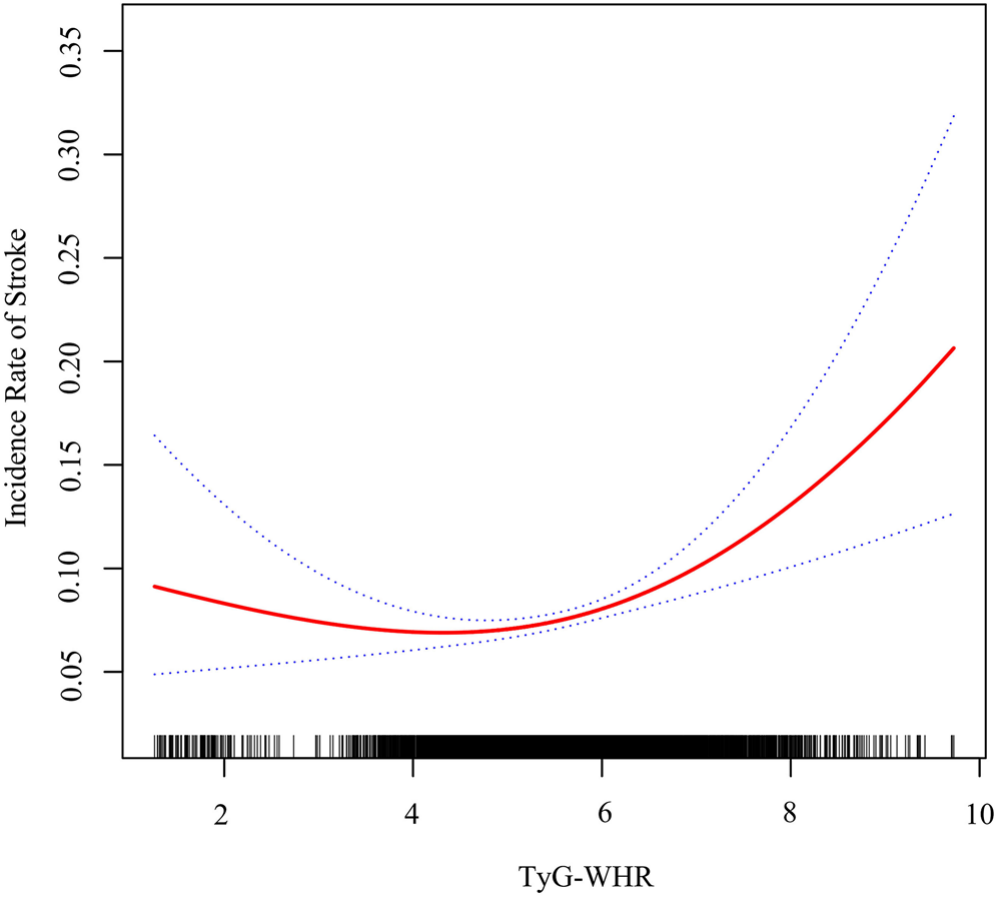
The nonlinear relationship between the TyG–WHR and stroke risk among all participants. TyG–WHR, triglyceride–glucose–waist-to-hip ratio.

The association between the TyG–WHR and stroke incidence was nonlinear through the application of a Cox proportional hazards regression model with cubic spline functions (Fig. 5) and a log-likelihood ratio test *p* < 0.05 (*p* = 0.002). Age, gender, geographic location, education, marital status, physical activity, cigarette smoking and alcohol consumption status, weight, SBP, DBP, HDL–C, and LDL–C were adjusted. The relationship between the TyG–WHR and stroke risk was nonlinear, with a TyG–WHR inflection point of 4.635 (Table 5). Postidentification, a two-piecewise Cox proportional hazards regression model was utilized to ascertain the HR and CI on either side of the demarcation. Table 5 shows that the increased likelihood of stroke was associated with the TyG–WHR on the left side of the inflection point (HR, 0.813; 95% CI, 0.662–0.999; *p* = 0.049), but the correlation was not significant. In contrast, the correlation was statistically significant on the right side of the inflection point (HR, 1.271; 95% CI, 1.131–1.429; *p* < 0.001).

**Table 5.** Results of the two-piecewise linear regression model

Outcome: incident stroke	HR (95% CI)	*p* value
Fitting model by standard linear regression	1.134 (1.028, 1.250)	0.0118
Inflection points of the TyG–WHR	4.635	
<4.635	0.813 (0.662, 0.999)	0.0491
≥4.653	1.271 (1.131, 1.429)	<0.0001
*p* for log-likelihood ratio test	0.002	
1.26 (1.15, 1.38)	1.24 (1.13, 1.36)	<0.0001

The data in this table were adjusted for age, gender, geographic location, education, marital status, physical activity, cigarette smoking and alcohol consumption status, weight, SBP, DBP, HDL–C and LDL–C. HR, hazard ratio; CI, confidence interval; TyG–WHR, triglyceride–glucose–waist-to-hip ratio.

**Figure 6.**
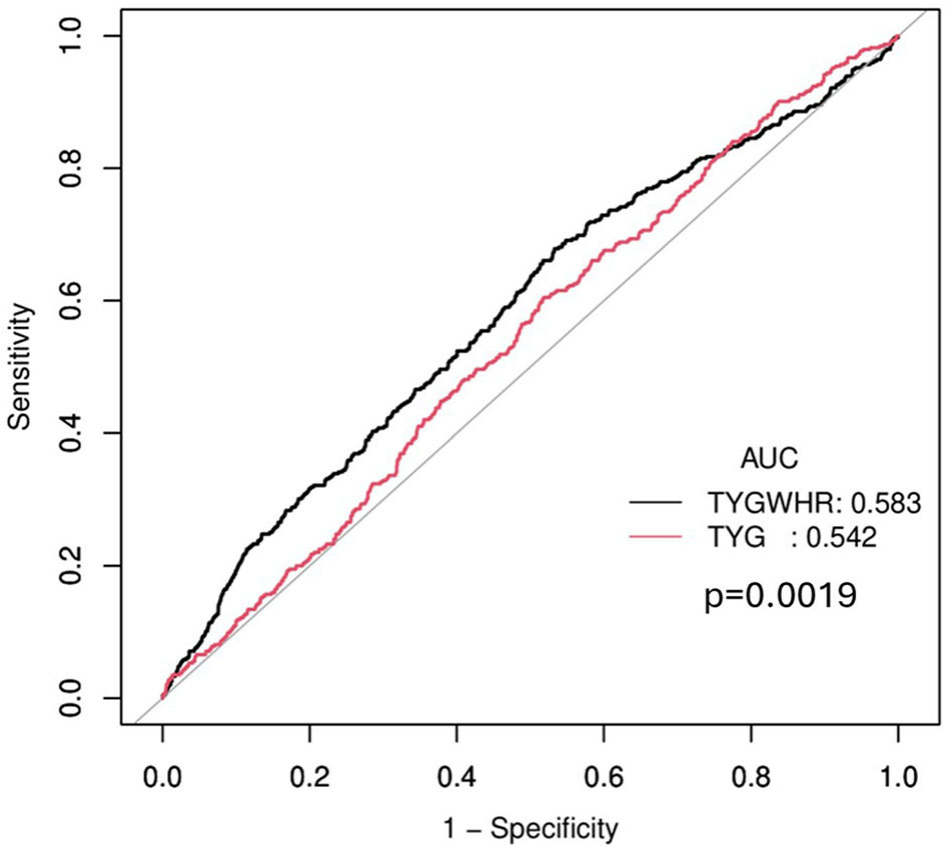
ROC curves comparing the predictive performance of the TyG–WHR and TyG indices for incident stroke. ROC, receiver operating characteristic; TyG, triglyceride–glucose; WHR, waist-to-hip ratio.

A comparative ROC analysis was conducted to assess the predictive performance enhancement of TyG–WHR compared with the TyG index. The AUC for TyG–WHR was 0.583, significantly surpassing that of TyG (AUC = 0.542; *p* = 0.002; Fig. 6). Furthermore, both the net reclassification improvement and integrated discrimination improvement for TyG–WHR over TyG were 0.057, indicating a modest enhancement in stroke risk classification beyond TyG alone.

### Results of subgroup analysis

**Figure 7.**
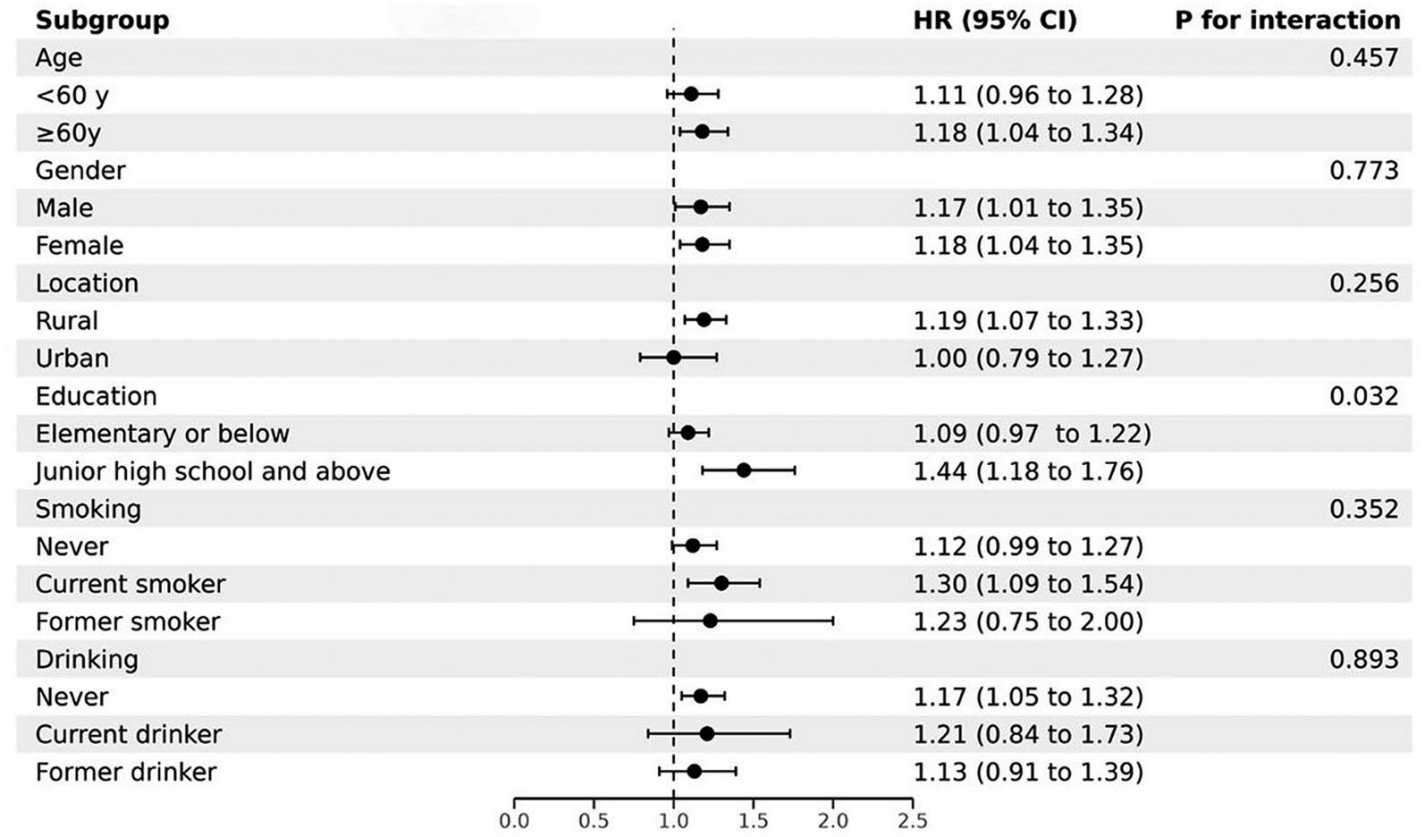
Forest plot of stratified analysis between the TyG–WHR and stroke by age, gender, geographic location, education, and cigarette smoking and alcohol consumption status. TyG–WHR, triglyceride–glucose–waist-to-hip ratio; HR, hazard ratio; CI, confidence interval.

Subgroup analysis was employed to investigate whether the association between TyG–WHR and incident stroke varied across key demographic and behavioral strata (Fig. 7). Stratification was conducted based on age, gender, place of residence, education level, marital status, and physical activity. The positive association between TyG–WHR and stroke remained generally consistent across these subgroups. However, interaction analysis revealed that education level significantly modified this relationship (*p* = 0.032), while no statistically significant interaction effects were observed for the other variables. These results suggest that the relationship between TyG–WHR and stroke is stable mainly across different population subgroups, potentially modifying educational attainment.

## Discussion

This prospective study investigated the relationship between the TyG–WHR and stroke. The results demonstrated a significant positive association between the TyG–WHR and stroke. This relationship was characterized by nonlinearity. In addition, an inflection point was identified, and different associations between the TyG–WHR and stroke risk were noted on both sides of the inflection point. The reliability of the results was confirmed by sensitivity and subgroup analyses.

The TyG index, which was initially proposed as a reliable surrogate marker for IR, has subsequently been shown to be closely associated with the development of stroke (Wang et al., 2021, 2023; Zhao et al., 2021; Xu et al., 2023; Yang et al., 2023; Yin et al., 2024). In addition, several studies have indicated a positive correlation between obesity and stroke. Obesity is divided into generalized and central obesity, with BMI representing the former and indicators, such as the WC, WHR, and WHtR, representing the latter. Central obesity is more strongly associated with visceral fat deposition (Wang et al., 2024). Huang et al. also reported that an elevated TyG and enlarged waist are associated with an elevated risk of stroke (Huang et al., 2023). The regional distribution of body fat is more important than obesity for the risk of cerebrovascular disease associated with body weight and excess adiposity. WHR, as a measure of central obesity, has been shown in a case-control study to be elevated as a risk factor for stroke (Namaganda et al., 2022). It has been shown that for every 10% increase in WHR, there is a 75% increase in the risk of aortic ischemic stroke (Marini et al., 2020). A prospective, multicenter, case-control study explored the association between obesity and early-onset cryptogenic ischemic stroke (CIS). Abdominal obesity, as measured by the WHR, was shown to be an independent risk factor for CIS in young adults after strict adjustment for concomitant risk factors (Jaakonmäki et al., 2022). The results in the current study indicated a positive association between stroke and the TyG–WHR. The participants with a lower TyG–WHR had a markedly lower stroke incidence compared with participants with a higher TyG–WHR. With an elevated TyG–WHR, the risk of stroke also increased.

A cohort study in the United States demonstrated that an elevated TyG index is strongly associated with an increased risk of stroke, with participants in the highest quartile having a higher risk of stroke compared with participants in the lowest quartile of the baseline TyG index (HR [95% CI] 1.254 [1.014–1.552]; Yin et al., 2024). Another cohort study from China also showed a 34% increase in stroke risk for every 1-SD increase in the TyG index (Wang et al., 2023). Jiang et al. (2024) reported that the TyG was linearly associated with the risk of stroke death. However, no report has investigated the relationship between the TyG–WHR and stroke risk. This study showed that the relationship between the TyG–WHR and stroke risk was nonlinear, with the inflection point of the TyG–WHR at 4.635. No significant correlation was detected between the TyG–WHR and stroke on the left side of the inflection point. Still, a significant correlation was detected on the right side of the inflection point. Stratification analysis did not identify confounding variables affecting the TyG–WHR and stroke association. The results of the current study may have important implications for clinical practice but still warrant further validations with additional samples.

The study reveals a nonlinear relationship between TyG–WHR and stroke, with a threshold of 4.635. The limited literature on this association suggests a critical point where IR and central adiposity significantly elevate cerebrovascular risk. Analysis in Table 5 shows a 27.1% higher stroke risk for each 1-SD increase in TyG–WHR above the inflection point (HR = 1.271; 95% CI, 1.131–1.429; *p* = 0.0001), while below it, the relationship is not statistically significant (HR = 0.813; 95% CI, 0.662–0.999; *p* = 0.0491). This indicates a potential metabolic threshold where protective mechanisms may weaken, increasing vascular vulnerability. Further research is needed to confirm this threshold's relevance across different populations. The study emphasizes the need for caution in interpretation and underscores TyG-WHR's importance as one of many potential indicators in stroke risk assessment.

The nonlinear relationship between TyG–WHR and stroke may indicate a threshold-dependent metabolic shift. At lower levels of IR and central adiposity, physiological compensatory mechanisms, such as insulin sensitivity in the skeletal muscle and hepatic lipid regulation, may mitigate cerebrovascular risk (Willette et al., 2015). However, beyond a critical threshold of TyG–WHR, increasing visceral fat and IR could synergistically exacerbate endothelial dysfunction, systemic inflammation, and thrombotic potential (La Rosa et al., 2025). These processes may drive the development of atherosclerosis, arterial stiffness, and cerebrovascular events (Westerbacka and Yki-Järvinen, 2002; Won et al., 2018). The plateau or inverse trend observed at lower TyG–WHR levels may suggest a saturation point where additional metabolic disturbances minimally affect stroke risk. Further research is necessary to confirm these hypotheses and investigate potential intermediary biomarkers.

Although the exact mechanisms of the relationship between the TyG–WHR and stroke are not fully understood, the link between obesity and elevated risk of stroke has been confirmed in numerous studies (Després, 2001; Ritchie and Connell, 2007; Horn et al., 2023). Specifically, abdominal or visceral obesity is associated with metabolic abnormalities, such as IR and dyslipidemia (Després, 2001; Ritchie and Connell, 2007). These metabolic disturbances lead to the development of atherosclerosis, which is a key factor in the occurrence of stroke (Huang et al., 2022; Zhou et al., 2022). It has also been shown that the relationship between the TyG index and stroke risk may be related to IR (Huang et al., 2022; Huo et al., 2024). IR is associated with the development of atherosclerosis, plaque rupture, and the formation of endothelial dysfunction (Guo et al., 2021). In addition, IR alters platelet function, leading to increased adhesion, activation, and aggregation, which may lead to arterial stenosis or obstruction and stroke (Du et al., 2014). Therefore, the mechanism underlying the relationship between the TyG–WHR and stroke incidence may be related to four factors (FPG, TG, WHR, and IR).

The current study had several strengths. First, data were used from the CHARLS database, a well-designed, highly representative database with a large sample size, reliable data, and a long follow-up period. Second, a prospective cohort study design was used, effectively capturing causality and reducing the effects of recall bias. Third, the Cox proportional risk model was used in data analysis to find a significant correlation between the TyG–WHR and stroke risk. The nonlinear relationship between the TyG–WHR and stroke was further investigated through smooth curve fitting and threshold effect analysis. In addition, comprehensive covariate adjustment was performed to ensure the robustness of the results. Stratified and sensitivity analyses confirmed the consistency of the findings across different populations. The combination of these strategies makes the study unique in revealing the relationship between the TyG–WHR and stroke risk. The TyG–WHR is a readily available marker, and a nonlinear relationship between the TyG–WHR and stroke was observed in the study. The inflection point of the TyG–WHR was 4.635. This finding may provide a basis for decision-making in clinical practice regarding stroke prevention.

However, the study had limitations. First, the participants were all from an older population in China (≥45 years of age), and caution was needed when extrapolating the findings to other ethnic and younger groups. Therefore, additional multicenter studies are warranted to validate the findings and further explore the applicability in different populations to determine the generalizability of the results. Second, as with all observational studies, residual confounding by unmeasured or uncontrolled confounders, such as a family history of stroke, diet, and indicators of inflammation, may remain despite adjustment for known potential confounders. Third, the diagnosis of stroke was based exclusively on self-report, which may lead to some small misclassification bias. Fourth, self-reported stroke diagnoses, especially among older participants, may introduce misclassification. This could lead to underreporting and weaken the observed associations, although such bias is likely nondifferential. Future research should focus on integrating clinically confirmed diagnoses. Lastly, this study's findings may not apply to younger populations or other ethnic groups due to the restricted study cohort of middle-aged and older Chinese adults. It is essential to conduct additional research in varied cohorts to validate these results across broader populations.

## Conclusions

In this prospective cohort study, we identified a nonlinear relationship between TyG–WHR and stroke incidence in middle-aged and elderly Chinese adults, with a discernible inflection point at 4.635. The results indicate that elevated TyG–WHR levels may signify an increased cerebrovascular risk. Although further research is required to validate the clinical significance of this threshold, our findings underscore the potential utility of TyG–WHR as a supplementary indicator for early risk assessment and preventive interventions in this demographic.

## Data Availability

The data that support the findings of this study are available from the corresponding author upon reasonable request.

## Synthesis

Reviewing Editor: James Howard, Brandeis University Decisions are customarily a result of the Reviewing Editor and the peer reviewers coming together and discussing their recommendations until a consensus is reached. When revisions are invited, a fact-based synthesis statement explaining their decision and outlining what is needed to prepare a revision will be listed below. The following reviewer(s) agreed to reveal their identity: NONE.

This study helps to link a relatively easily measurable physiological parameter to stroke risk in a large sample of older Chinese participants. This is a good first step toward developing a non-invasive treatment that may reduce the risk of stroke more broadly. The list of suggestions and comments is as follows:

1. In the Methods section, please list the population breakdown in terms of gender.

2. Did the TyG-WHR change the over the course of the data collection process? If so, how do those changes correlate with a change in stroke incidence?

3. In general, the figure and table legends need to have more information. All symbols and abbreviations depicted in the figures and tables should be explained in the figure legends.
